# Child and Adolescent Mental Health Policy in Low- and Middle-Income Countries: Challenges and Lessons for Policy Development and Implementation

**DOI:** 10.3389/fpsyt.2020.00150

**Published:** 2020-03-18

**Authors:** Wei Zhou, Feiyun Ouyang, Oyun-Erdene Nergui, Joseph Benjamin Bangura, Kwabena Acheampong, Isaac Yaw Massey, Shuiyuan Xiao

**Affiliations:** ^1^Xiangya School of Public Health, Central South University, Changsha, China; ^2^Hospital Administration Institute, Xiangya Hospital, Central South University, Changsha, China; ^3^Department of Psychiatry, The Second Xiangya Hospital, Central South University, Changsha, China

**Keywords:** children and adolescents, mental health policy, low- and middle-income countries, challenges and barriers, policy development and implementation

## Abstract

**Background:** Child and adolescent mental health (CAMH) policy is essential for the rational development of mental health systems for children and adolescents. However, there is a universal lack of CAMH policy, especially in low- and middle-income countries (LMICs). Therefore, this review aims to identify challenges and lessons for LMICs to develop and implement CAMH policy.

**Methods:** PubMed (1781-), MEDLINE (1950-), EMBASE (1966-), and PsycINFO (1895-) were searched from inception to December 31, 2018, for publications on CAMH policy development and/or implementation. Abstracts and main texts of articles were double screened, and extracted data were analyzed through thematic synthesis.

**Results:** A total of 31 publications were included through the systematic review. Six major challenges were identified for CAMH policy in LMICs: (i) poor public awareness and low political willingness; (ii) stigma against mental disorders; (iii) biased culture values toward children, adolescents and CAMH, from developmental nihilism to medicalization; (iv) the lack of CAMH data and evidence, from service statistics to program evaluation; (v) the shortage of CAMH resources, including human resources, service facilities, and funding; and (vi) unintended consequence of international support, including reducing local responsibilities, planning fragmentation, and unsustainability. Six lessons to overcome challenges were summarized: (i) rethinking the concept of CAMH, (ii) encouraging a stand-alone CAMH policy and budget, (iii) involving stakeholders, (iv) reinforcing the role of research and researchers in policy process, (v) innovating the usage of human and service resources, and (vi) maximizing the positive influence of international organizations and non-governmental organizations.

**Conclusion:** Many LMICs are still facing various challenges for their CAMH policy development and implementation. To overcome the challenges, great and long-term efforts are needed, which include great determination of from domestic and global agents, multidisciplinary innovations, and collaboration and coordination from different sectors.

## Introduction

Children and adolescents constitute 44% of the world's population (1), and 10–20% of them suffer mental health problems (2), which is a leading cause of health-related burden in this age group (3). As the majority of adult mental disorders have their first onset in childhood and adolescence (4), prevention and treatment for children and adolescents are of significant importance.

Child and adolescent mental health (CAMH) policy, as a roadmap, guides the development of CAMH services (5, 6). Fully realizing the importance of CAMH policy, the World Health Organization (WHO) in 1977 recommended that every country throughout the world should have a National Plan for Child Mental Health. In 1992, the International Association for Child and Adolescent Psychiatry and Allied Profession endorsed the WHO's 1977 recommendation (7). In 2005, WHO released *Mental Health Policy and Service Guidance Package: Child and Adolescent Mental Health Policies and Plans* to help nations develop CAMH policy (8).

However, Shatkin and Belfer's (5) survey from 2002 found that no countries in the world had a mental health policy or action plan uniquely pertaining to children and adolescents and that only 18% countries (35 of 191) had mental health policies, which might have some beneficial impact on children and adolescents. The WHO Child Mental Health Atlas published in 2005 also demonstrated the paucity of CAMH policy, as only 30% of the 66 reported countries had national CAMH policy (9, 10). The global absence of CAMH policy remains unchanged. According to WHO Mental Health Atlas 2017, only 46% of 78 responding countries had a plan or strategy for CAMH (11).

Approximately 90% of children and adolescents live in low- and middle-income countries (LMICs), where they form up to 50% of the population (1). However, results from Shatkin and Belfer's survey and WHO Child Atlas Project consistently revealed that the dearth of CAMH policy in LMICs was even more severe (5, 9, 10). Based on the above fact, this article focuses on LMICs according to the latest classification by World Bank (12). Through a systematic review, this study aims to identify (i) challenges for LMICs to develop and implement CAMH policy and (ii) lessons for LMICs to overcome challenges, based on a global-scope experience.

## Methods

Based on the theory of policy life circle (13) and suggestions of *WHO Mental Health Policy and Service Guidance Package* (14), we divided the whole policy process into three stages: policy development, implementation, and outcome. As the policy outcome or the progress of policy is a result of policy development and implementation, it will be more useful to examine the stages of policy development and implementation to find the reasons for unsatisfactory policy outcome. Therefore, we focused on exploring challenges at the first two policy stages of CAMH policy in LMICs.

### Search Strategy and Selection Criteria

We conducted a systematic review using the steps recommended in the PRISMA 2009 Checklist. PubMed (1781−), MEDLINE (1950−), EMBASE (1966−), and PsycINFO (1985−) were searched from inception to December 31, 2018, by WZ on January 4, 2019. Searches in the field of title were conducted using the following terms: (“child” or “children” or “adolescent” or “youth” or “young people”) AND (“psychiatry” or “psychiatric” or “mental health” or “psychology” or “psychological”) AND (“policy” or “policies” or “plan” or “strategy” or “strategies” or “legislation” or “law”). Bibliographies were also hand-searched to identify other relevant publications. Publication language was restricted to English.

We included both original research and reviews on CAMH policy development or implementation. Studies were excluded if they (i) were descriptive introductions on content of CAMH policies and lacked information on policy development or implementation; (ii) were only data analysis to examining the progress of certain CAMH policy goal, without discussion of policy challenges or lessons; (iii) were CAMH-related research, which provided evidence for treatment or implications for future CAMH policies; (iv) introduced a new instrument for CAMH policy development or evaluation, but without empirical information on CAMH policies in LMICs for data extraction; (v) were book reviews or conference abstracts; or (vi) were not available in full text.

### Screening, Abstraction, and Synthesis

Identified citations went through title and abstract screening and then full-text screening. The two-stage screening was conducted independently by WZ and FO, and any disagreement was resolved through consensus.

We extracted data from included publications, which included countries/regions being studied, and sentences or paragraphs on LMICs' challenges and global lessons for CAMH policy development and implementation. Extracted data were analyzed with thematic synthesis. The process of synthesis went through the following steps: (i) familiarization with data, (ii) generation of initial codes, (iii) searching for themes, (iv) review of themes, and (v) definition and naming of themes (15, 16). The process was iteration, and these steps were not unidirectional.

## Results

**Figure 1:**
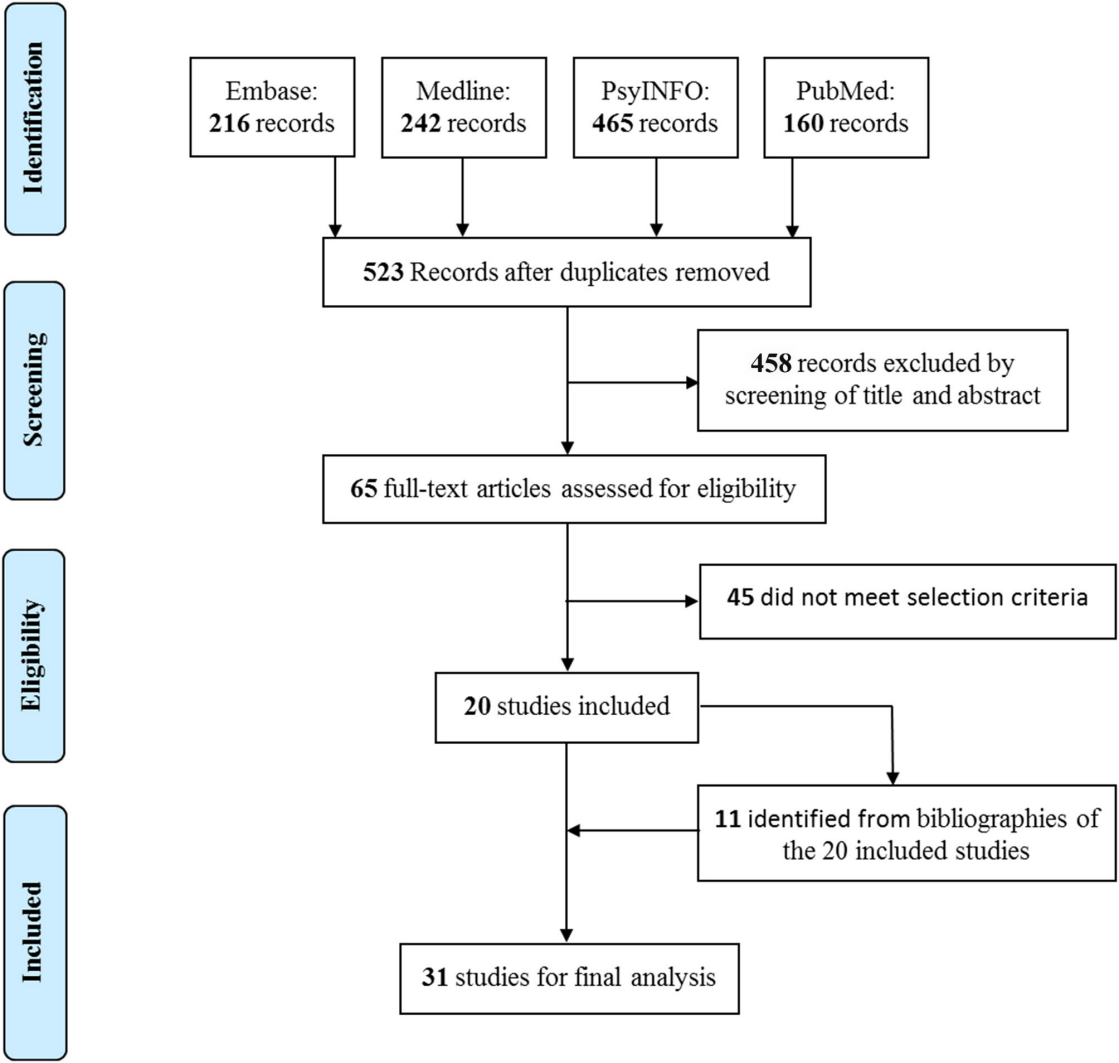
Process and results of study selection.

In this systematic review, 523 records were initially identified from the four databases, and 20 met the selection criteria. Based on the reference lists of the 20 included publications, we further identified 11 eligible studies for the final analysis after assessing full texts (Figure 1). Among the 31 included publications (Appendix 1), seven focused on specific countries or regions of low-middle-income economy, including Ghana, India, Iraq, South Africa, Uganda, Vietnam, Zambia, Latin America and the Caribbean, and sub-Saharan Africa; two restricted the discussion on developing countries or resource-poor countries; 14 were based on countries or regions of high-income economy, including Canada, Hungary, Lithuania, United Kingdom, United States, and Europe; and the remaining eight elaborated the issue of CAMH policy in general or globally.

### Challenges for LMICs' CAMH Policy Development and Implementation

Based on literature on LMICs or regions, we found that LMICs are faced with not only the absence of CAMH policy, but also implementation difficulties, including the lack of feasibility and sustainability of policy (17, 18). Six major challenges for LMICs' CAMH policy emerged as themes from thematic analysis. The detailed list of codes and themes is presented in Appendix 2.

#### Poor Public Awareness and Low Political Willingness

Public awareness and political willingness are key factors determining whether CAMH can enter policy agenda. However, even compared with adult mental health, CAMH is neither understood or interested by the public, nor placed as a priority among policy makers in LMICs (18–20). This is because CAMH is a field with relatively new development of knowledge, and the dissemination of knowledge is slower (5, 9, 17). Therefore, the magnitude of CAMH problem is not recognized by the public or policy makers (20). Moreover, many LMICs are confronted with political turmoil, economic upheavals, and other health challenges including HIV/AIDS, tuberculosis, and high infant and maternal mortality (8, 17, 20–23), which largely distract the public's and politicians' attention from CAMH. The situation is worsened by the facts that CAMH professionals are reluctant to engage in policy debates (5) and that children and adolescents are least capable of advocating for themselves, as they cannot vote, have almost no political or economic influence, and are even often abrogated rights (5, 8, 9).

#### Stigma Against Mental Disorders

It is well known that stigma widely exists in patients with mental disorders, and children and adolescents are particularly vulnerable to stigma. It can negatively affect children and adolescents and their families obtaining support and seeking for mental health services, as parents are often blamed and even forced to move frequently because of mental health problems of their children (8, 24). Stigma of mental disorders is also reported among health professionals. Some young professionals in LMICs like Vietnam and Sierra Leone are reluctant to take CAMH as their career and service providers are sometimes discriminated for working in CAMH facilities (24, 25). Stigmatizing views are also found in policy makers. For example, in a quoted interview script of a Uganda politician, mental disorder was described as a bad disease with personal hate, and no interest in mental health was also clearly expressed (17). The widespread stigma makes stakeholders reluctant to talk the issue of CAMH in public forums, which turns CAMH, an important issue, into a low-profile topic in policy agenda.

#### Biased Culture Values Toward Children and Adolescents and CAMH

There are several perceptions toward children and adolescents (including CAMH) embedded in different cultures in LMICs. Relevant perceptions were well summarized by Harper and Çetin (19): (i) children belong to their parents, and society cannot interfere; (ii) children's development is determined by heredity, fate, or immutable social facts such as race, class, or social group, and efforts for change are worthless; (iii) schools suffice for child development, including CAMH, and no other extra attention and arrangement are needed; (iv) CAMH is seen as a luxury, and mental health need among children and adolescents is less important than physical health; (v) CAMH problems are treated as a pure medical issue, without recognizing the role of nonmedical factors; (vi) children and adolescents are regarded as an economic investment, and policy's economic benefits are emphasized over other aspects such as children's rights; (vii) children and adolescents' rights should be recognized. The first six perceptions either hold an evasive, passive attitude or suggest inaction toward CAMH, or adopt a unilateral view for CAMH, which all exert negative influence on CAMH policy development and implementation.

#### The Lack of CAMH Data and Evidence

Data and evidence are the foundation of policy formulation and evaluation. However, methodologically and culturally appropriate epidemiological studies of the prevalence of CAMH problems are few in most LMICs (2, 4, 5), which makes it difficult to determine the arrangement of CAMH services in both quantity and organizing manner. As for the statistics of current CAMH services in LMICs, the low responding rates of WHO CAMH Atlas survey (66 of 192) (9, 10) and WHO Mental Health Atlas 2017 (78 of 192) (11) indicate the difficulty in obtaining data, which was also encountered in the multicountry survey designed for our study (Supplementary Material). Evidence for effective treatment protocols and health interventions is also limited, and evaluation of CAMH policy or programs is not common (6, 8, 17, 23).

#### The Shortage of CAMH Resources

Mental health resources, including mental health services, human resources, and funding, are insufficient in LMICs (2, 26). Despite the large population of children and adolescents in LMICs, resources for CAMH are scarcer, compared with those for adults. According to WHO Mental Health Atlas 2017, the median number of mental health beds per 100,000 population in low-income and lower middle-income countries is below 7 for adults and below 0.2 for children and adolescents; the median number of psychiatrists per 100,000 population in LMICs is below 2 for adults and below 0.1 for children and adolescents (11). In addition to the quantity of human resources, capacity is also listed as a barrier, which includes capacity of decision makers to develop a feasible CAMH policy, capacity of service providers to implement CAMH program, and capacity of researchers to provide evidence (8, 17, 18). As for funding, most LMICs do not have a specific budget for CAMH, and paying CAMH services out of patients' pocket is very common (7, 27). In addition, sustainability of funding is affected by factors such as election cycles (8).

#### Unintended Consequence of Support From International Organizations and Non-governmental Organizations

International organizations (IOs) and nongovernmental organizations (NGOs) play an important role in promoting CAMH in LMICs, through providing expertise, funding, and/or services (8, 23, 25, 28). However, support from IOs and NGOs also can have unintended consequences. First, the presence of support from IOs and NGOs may reduce local government's sense of urgency for taking responsibility of CAMH (25). Second, driven by donors' interest, many IOs and NGOs implement programs targeting at specific disorders, such as autism (23). For LMICs relying heavily on external support for CAMH, disorder-specific orientation may lead to local government's biased focus on certain aspects of CAMH and fragmentation of policy planning (6, 23, 28).Third, support from IOs and NGOs tends to be project-oriented with short time term. In some LMICs, where most CAMH services are provided by IOs and NGOs, the cease of projects will disturb children's and adolescents' utilization of mental health services (8, 28).

### Overcoming Challenges: Global Experience and Lessons

Six major lessons to overcome challenges emerged as themes from thematic analysis on the global-scope literature. The detailed list of codes and themes is presented in Appendix 3.

#### Rethinking the Concept of CAMH

In LMICs such as Nigeria, CAMH problems are regarded as “a curse by the gods and punishment for evil doing,” according to local beliefs (20). Therefore, it is very necessary to rethink local perception of CAMH problems, which is good for reducing stigma and raising public awareness and political willingness. Globally, CAMH has been described in several ways. For example, to promote the public's acceptability and the parity between mental and physical health, CAMH problems are framed as being like any other physical illnesses (29). For another example, policy documents of England describe CAMH as a socioeconomic issue. Child and adolescent mental health problem is viewed as consequence of socioeconomic inequities and difficulties and as socioeconomic burden in the future (30). More recently, positive mental health is proposed, which emphasizes that mental health is not only the absence of mental disorders but also positive child development (8, 31, 32). Scientific evidence and social and cultural context should be considered when thinking CAMH in LMICs.

#### Encouraging a Stand-Alone CAMH Policy and Budget

Although CAMH can be covered in policies of education, social welfare, and general health, a specific CAMH policy is still needed (33). On one hand, having a specific CAMH policy in place can demonstrate the government's commitment (34). Once government is committed to CAMH, the challenge of lacking resources can also be alleviated through resource mobilization by the government. Under a specific CAMH policy, a stand-alone budget should be assigned accordingly, so as to avoid competition from other health issues and even from adult mental health (32, 35). On the other hand, because CAMH should be considered in a wider social context and because CAMH services are also intersectorial in nature (6, 7), a specific CAMH policy can provide an overall framework to guide intersectorial collaboration.

#### Involving Stakeholders

Similar to mental health policy in general, full involvement of stakeholders is needed (27, 36). A consensus among stakeholders through negotiation will lay the foundation for CAMH policy's successful implementation (27). Stakeholders include mental health professionals, parents, social services, religious leaders, and educators (19). Moreover, specifically to CAMH policy, children and adolescents should not be regarded only as passive recipient of protection and intervention by adults; instead, they need to be recognized with respect and given a voice during policy formulation (8, 19).

#### Reinforcing the Role of Research and Researchers in Policy Process

Because of status quo that children and adolescents have weak or even no voice in society, researchers should take the responsibility of advocacy through research and involving policy process (20, 37). First, methodologically sound and culturally compatible research on CAMH prevalence, treatment, and strategy evaluation should be conducted to make the problem of CAMH visible and to provide solutions, which can be adopted in policy and service planning. However, it should be well noted that merely providing data and generating evidence are far from enough (38). Researchers should understand policy process and communicate CAMH evidence to policy makers and the public in a more user-friendly way. For example, syntheses of research evidence, rather than scattered knowledge, are preferred (37, 39, 40). Meanwhile, partnership between researchers and policy makers is suggested, for timely communication of research findings. To further prioritize CAMH into policy agenda, some literature suggests that researchers can point out the relationship between CAMH and other key issues in LMICs (8). In China, scholars linked the case management of patients with psychotic disorders to social stability and then successfully persuaded Chinese government to launch a series of policies and fund National Continuing Management and Intervention Program for Psychosis (41). As poverty is an important issue in many LMICs, advocacy through linking CAMH with local economy such as quality of future labor forces and analyzing economic impact of CAMH problems could be useful attempts (28, 30).

#### Innovating the Usage of Human and Service Resources

As the shortage of CAMH resources in LMICs is difficult to change in the short term (42), it will be more feasible to make innovative and optimal use of the existing human and service resources (18). First, CAMH training can be provided to pediatricians, primary healthcare providers, and school teachers, so that they can deliver CAMH screening and less complex interventions (18, 23). Second, collaboration with other systems should be encouraged (23). Those systems include health and non-health ones, such as HIV/AIDS project networks, education systems, and faith-based organizations. Collaboration systems can be formal and informal ones, such as traditional healers (8). Third, capacity-building programs for service users should be developed. Some effective programs include parenting skills training for families and life skills training for children and adolescents themselves (18). Those programs can relieve CAMH problems from the perspective of prevention and then reduce pressure on CAMH medical resources, such as pediatric psychiatrists and hospitals.

#### Maximizing Positive Influence of IOs and NGOs

Despite some unintended consequences of IOs' and NGOs' work, their role in promoting CAMH policy and services is undeniably important in both high-income countries (HICs) and LMICs (23, 43). They can enhance their positive influence in the following ways: first, intergovernment organizations, such as the United Nations (UN) and WHO, can initiate international treaties to promote LMICs' governments' commitment to CAMH (8). From the 30-year-ago *UN Convention of the Rights of the Child* to the recent *Comprehensive Mental Health Action Plan 2013–2020*, CAMH is written into member states' obligations. Second, coordination between local governments, IOs, and NGOs can reduce competitions, overlapping of resource, and fragmented planning of CAMH (28). Third, IOs and NGOs should consider sustainability at the beginning of their CAMH program development and naturally move programs' independence and sustainability (25). In addition, instead of leading CAMH services themselves, IOs and NGOs should make more efforts on supporting local professionals, through providing consultation, capacity building, and funding research (19, 44).

## Discussion

Although CAMH policy is an important issue in LMICs, there is very limited research on this topic, as this systematic review found only nine publications on CAMH policy in LMICs or regions of low- to middle-income economy. It indicates that CAMH policy is not only neglected by the public and policy makers, but also receives insufficient attention in the academic circle. Our research results suggest that research is needed not only for raising public awareness, but also for providing local experience and expertise for CAMH policy formulation and implementation and promoting CAMH policy progress in LMICs. Therefore, more research on this topic should be advocated in the future.

Many challenges presented in the results part can be commonly found in HICs' or LMICs' mental health policy in general (42); however, when they come to CAMH policy, they have greater severity. One potential reason is that the mental health needs of children and adolescents are often not considered separately from those of adults, especially under resource-constraint settings. For example, many LMICs do not set mental health professionals or facilities specifically for children and adolescents (7, 18, 22). Because of different developmental competencies and growth trajectories, mental health professionals without special training for CAMH cannot provide mental health services needed by children and adolescents. This makes CAMH resources extremely insufficient. Furthermore, children and adolescents are vulnerable groups with weak voices in political and economic arenas (5, 8, 9). As a result, their needs are more likely to be neglected, compared with adult patients with mental disorders.

Many challenges, such as the lack of CAMH resources, also exist in HICs (30, 32, 43), and HICs have their thinking and proposed solutions, some of which are included in this review. We acknowledge the difference of socioeconomic and cultural context between HICs and LMICs (18) and potential problems of direct transplantation of HICs' experience (45). Therefore, we did not present specific services organizing models, but only selected general lessons, which may be applicable for or can provide inspiration for LMICs. For example, the holistic approach to CAMH was not included, because the literature shows that even HICs such as Lithuania faced many challenges and obstacles in its implementation (31). To better solve challenges for LMICs, more research based on local experience and expertise is in extreme need.

This review was limited to English language publications, which potentially excluded some relevant studies on non–English-speaking countries. In addition, there were few available studies on CAMH policy in LMICs; therefore, there might be some other challenges for CAMH policy in LMICs unreported in existing publications, which were also not captured in our review. In an attempt to cross-check the representativeness of the challenges identified by our systematic review, we also designed an exemplifying multicountry survey to cross-check our results, based on a convenient sampling of PhD students from six LMICs. The survey results were consistent with findings from our systematic review. Survey details were presented as a Supplementary Material.

## Conclusion

There are many challenges for LMICs to develop and implement CAMH policy. Overcoming all the challenges will be a long and difficult process. In the future, promoting CAMH policy in LMICs will need great determination from domestic and global agents, multidisciplinary innovations, and collaboration and coordination from different sectors.

## Data Availability Statement

The datasets generated for this study are available on request to the corresponding author.

## Author Contributions

WZ and SX designed this research, including the systematic review protocol and questionnaire for the multi-country survey. WZ and FO screened for inclusion papers in the systematic review. FO, O-EN, JB, KA, and IM collected data in the multi-country survey. WZ and SX extracted and analyzed data. WZ drafted the manuscript. All authors read and approved the final manuscript.

### Conflict of Interest

The authors declare that the research was conducted in the absence of any commercial or financial relationships that could be construed as a potential conflict of interest.

## References

[1] United Nations World Population Prospects: The 2010 Revision. New York, NY: United Nations (2011).

[2] Kieling C Baker-Henningham H Belfer M Conti G Ertem I Omigbodun O. Child and adolescent mental health worldwide: evidence for action. Lancet. (2011) 378:1515–25. 10.1016/S0140-6736(11)60827-1 22008427

[3] GBD 2016 Causes of Death Collaborators Global, regional, and national age-sex specific mortality for 264 causes of death, 1980-2016: a systematic analysis for the Global Burden of Disease Study 2016. Lancet. (2017) 390:1151−210. 10.1016/S0140-6736(17)32152-9 28919116 PMC5605883

[4] Patel V Flisher AJ Hetrick S McGorry P. The mental health of young people: a global public health challenge. Lancet. (2007) 369:1302–13. 10.1016/S0140-6736(07)60368-7 17434406

[5] Shatkin JP Belfer ML The global absence of child and adolescent mental health policy. Child Adolesc Mental Health. (2004) 9:104–8. 10.1111/j.1475-3588.2004.00090.x 32797484

[6] Belfer ML. Critical review of world policies for mental healthcare for children and adolescents. Curr Opin Psychiatr. (2007) 20:349–52. 10.1097/YCO.0b013e3281bc0cf4 17551349

[7] Sharan P Sagar R Mental health policy for children and adolescents in developing countries. J Ind Assoc Child Adolesc Mental Health. (2007) 3:1–4.

[8] WHO Child and Adolescent Mental Health Policies and Plans. Mental Health Policy and Service Guidance Package. Geneva: WHO (2005).

[9] WHO Atlas: Child and Adolescent Mental Health Resources: Global Concern: Implications for the Future. Geneva: WHO (2005).

[10] Belfer ML Saxena S. WHO child atlas project. Lancet. (2006) 367:551–2. 10.1016/S0140-6736(06)68199-3 16488783

[11] WHO Mental Health Atlas 2017. Geneva: World Health Organization (2018).

[12] World Bank Country and Lending Groups (2018). Available online at: https://datahelpdesk.worldbank.org/knowledgebase/articles/906519 (accessed January 3, 2020).

[13] Parsons W Public Policy: An Introduction to the Theory and Practice of Policy Analysis. Aldershot: Edward Elgar (1995).

[14] WHO WHO Mental Health Policy and Service Guidance Package: Monitoring and Evaluation of Mental Health Policies and Plans. Geneva: World Health Organization (2007).

[15] Thomas J Harden A. Methods for the thematic synthesis of qualitative research in systematic reviews. BMC Med Res Methodol. (2008) 8:45. 10.1186/1471-2288-8-45 18616818 PMC2478656

[16] Braun V Clarke V Using thematic analysis in psychology. Qual Res Psychol. (2006) 3:77–101 10.1191/1478088706qp063oa

[17] Mokitimi S Schneider M de Vries PJ. Child and adolescent mental health policy in South Africa: history, current policy development and implementation, and policy analysis. Int J Mental Health Syst. (2018) 12:36. 10.1186/s13033-018-0213-3 29983735 PMC6019826

[18] Kleintjes S Lund C Flisher AJ MHaPP Research Programme Consortium. A situational analysis of child and adolescent mental health services in Ghana, Uganda, South Africa and Zambia. Afr J Pyschiatry. (2010) 13:132–9 10.4314/ajpsy.v13i2.54360 20473475

[19] Harper G Çetin FC. Child and adolescent mental health policy: promise to provision. Int Rev Psychiatr. (2008) 20:217–24. 10.1080/09540260802030559 18569174

[20] Omigbodun O. Developing child mental health services in resource-poor countries. Int Rev Psychiatr. (2008) 20:225–35. 10.1080/09540260802069276 18569175

[21] Atilola O. Child mental-health policy development in sub-Saharan Africa: broadening the perspectives using Bronfenbrenner's ecological model. Health Promot Int. (2017) 32:380–91. 10.1093/heapro/dau065 25107920

[22] Al-Obaidi AK Budosan B Jeffrey L Child and adolescent mental health in Iraq: current situation and scope for promotion of child and adolescent mental health policy. Intervention. (2010) 8:40–51. 10.1097/WTF.0b013e3283387adf

[23] Belfer ML Rohde LA. Child and adolescent mental health in Latin America and the Caribbean: problems, progress, and policy research. Rev Panam Salud Publica. (2005) 18:359–65. 10.1590/S1020-49892005000900016 16354433

[24] Yoder HN Tol WA Reis R de Jong JT. Child mental health in Sierra Leone: a survey and exploratory qualitative study. Int J Mental Health Syst. (2016) 10:48. 10.1186/s13033-016-0080-8 27354854 PMC4924306

[25] Weiss B Dang HM Trung LT Sang DL Ngo VK Pollack A. A model for sustainable development of child mental health infrastructure in the LMIC world: Vietnam as a case example. Int Perspect Psychol Res Pract Consult. (2012) 1:63–77. 10.1037/a0027316 24701368 PMC3971880

[26] Saxena S Thornicroft G Knapp M Whiteford H. Resources for mental health: scarcity, inequity, and inefficiency. Lancet. (2007) 370:878–89. 10.1016/S0140-6736(07)61239-2 17804062

[27] Russell PS Mammen P Nair MK Russell S Shankar SR. Priority mental health disorders of children and adolescents in primary-care pediatric setting in India 1: developing a child and adolescent mental health policy, program, and service model. Ind J Pediatr. (2012) 79(Suppl. 1):S19–26. 10.1007/s12098-011-0426-9 21660409

[28] WHO Caring for Children and Adolescents with Mental Disorders: Setting WHO Directions. Geneva: World Health Organization (2003).

[29] Millard C Wessely S. Parity of esteem between mental and physical health. BMJ. (2014) 349:g6821. 10.1136/bmj.g6821 25398394

[30] Callaghan JE Fellin LC Warner-Gale F. A critical analysis of child and adolescent mental health services policy in England. Clin Child Psychol Psychiatr. (2017) 22:109–27. 10.1177/1359104516640318 27052891

[31] Kišunaite A Puras D Towards a holistic approach to children's rights in Lithuanian mental health policy: a case study. Arch Psychiatr Psychother. (2016) 2:40–7. 10.12740/APP/63231

[32] Braddick F Carral V Jenkins R Jane-Llopis E Child and Adolescent Mental Health in Europe: Infrastructures, Policy and Programmes. Luxembourg: European Communities (2009).

[33] Lourie IS Hernandez M A historical perspective on national child mental health policy. J Emot Behav Disord. (2003) 11:5 10.1177/106342660301100102

[34] Kutcher S Hampton MJ Wilson J. Child and adolescent mental health policy and plans in Canada: an analytical review. Can J Psychiatr. (2010) 55:100–7. 10.1177/070674371005500206 20181305

[35] Vanesa CB Fleur B Eva J Rachel J Dainius P Child and adolescent mental health policies, programmes and infrastructures across Europe. Int J Mental Health Promot. (2010) 12:10–26 10.1080/14623730.2010.9721822

[36] Zhou W Yu Y Chen L Xiao S. Evaluating China's mental health policy on local-level promotion and implementation: a case study of Liuyang Municipality. BMC Public Health. (2019) 19:24. 10.1186/s12889-018-6315-7 30616607 PMC6323835

[37] Knitzer J. Mental health services to children and adolescents: a national view of public policies. Am Psychol. (1984) 39:905–911. 10.1037/0003-066X.39.8.905 6476584

[38] Heflinger CA Dokecki PR The use of mental health standards in child and adolescent programs: what factors influence policy development and implementation? In: Paper Presented at the Annual Convention of the American Psychological Association. Los Angeles, CA (1985).

[39] Waddell C Lavis JN Abelson J Lomas J Shepherd CA Bird-Gayson T. Research use in children's mental health policy in Canada: maintaining vigilance amid ambiguity. Soc Sci Med. (2005) 61:1649–57. 10.1016/j.socscimed.2005.03.032 16029772

[40] Waddell C Offord DR Shepherd CA Hua JM McEwan K. Child psychiatric epidemiology and Canadian public policy-making: the state of the science and the art of the possible. Can J Psychiatr. (2002) 47:825–32 10.1177/070674370204700903 12500752

[41] Liu J Ma H He YL. Mental health system in China: history, recent service reform and future challenges. World Psychiatr. (2011) 10:210–6 10.1002/j.2051-5545.2011.tb00059.x 21991281 PMC3188776

[42] Knapp M Funk M Curran C Prince M Grigg M McDaid D. Economic barriers to better mental health practice and policy. Health Policy Plan. (2006) 21:157–70. 10.1093/heapol/czl003 16522714

[43] Kapócs G Balázs P The health policy in child and adolescent psychiatry in Hungary: a review of recent developments. New Med. (2017) 21:14–20. 10.5604/01.3001.0009.7843

[44] Turner J Pigott H Tomlinson M Jordans MJ. Developmental assistance for child and adolescent mental health in low- and middle-income countries (2007-2014): Annual trends and allocation by sector, project type, donors and recipients. J Glob Health. (2017) 7:020901. 10.7189/jogh.07.020901 29302326 PMC5737098

[45] Zhou W Yu Y Yang M Chen L Xiao S. Policy development and challenges of global mental health: a systematic review of national-level mental health policy studies. BMC Psychiatr. (2018) 18:138. 10.1186/s12888-018-1711-1 29776356 PMC5960139

