# Supplementary material for: Child and Adolescent Mental Health Policy in Low- and Middle-Income Countries: Challenges and Lessons for Policy Development and Implementation
Source: Front Psychiatry. 2020 Mar 18;11:150. doi: 10.3389/fpsyt.2020.00150 (PMC7094177; doi:10.3389/fpsyt.2020.00150)
Supplement: Supplementary file 1 [file Data_Sheet_1.docx]

**Appendix 1 The list of included studies**

1. Russell, P.S., et al., Priority mental health disorders of children and adolescents in primary-care pediatric setting in india 1: Developing a child and adolescent mental health policy, program, and service model. Indian Journal of Pediatrics, 2012. 79(SUPPL. 1): p. S19-S26.
2. Harper, G. and F.C. Çetin, Child and adolescent mental health policy: Promise to provision. International Review of Psychiatry, 2008. 20(3): p. 217-224.
3. Sharan, P. and R. Sagar, Mental health policy for children and adolescents in developing countries. Journal of Indian Association for Child and Adolescent Mental Health, 2007. 3(1): p. 1-4.
4. Mokitimi, S., M. Schneider, and P.J. de Vries, Child and adolescent mental health policy in South Africa: history, current policy development and implementation, and policy analysis. International Journal Of Mental Health Systems, 2018. 12: p. 36-36.
5. Atilola, O., Child mental-health policy development in sub-Saharan Africa: broadening the perspectives using Bronfenbrenner's ecological model. Health Promotion International, 2017. 32(2): p. 380-391.
6. Al-Obaidi, A.K., B. Budosan, and L. Jeffrey, Child and adolescent mental health in Iraq: current situation and scope for promotion of child and adolescent mental health policy. Intervention (15718883), 2010. 8(1): p. 40-51.
7. Funk, M., et al., Child and Adolescent Mental Health Policies and Plans. Mental Health Policy and Service Guidance Package. 2005, Geneva: WHO.
8. Belfer, M.L. and L.A. Rohde, Child and adolescent mental health in Latin America and the Caribbean: problems, progress, and policy research. Salud mental de niños y adolescents en América Latina y el Caribe: problemas, avances e investigación en políticas., 2005. 18(4/5): p. 359-365.
9. Shatkin, J.P. and M.L. Belfer, The Global Absence of Child and Adolescent Mental Health Policy. Child & Adolescent Mental Health, 2004. 9(3): p. 104-108.
10. World Health Organization. Atlas: child and adolescent mental health resources: global concern: implications for the future. Geneva: WHO; 2005
11. Belfer ML. Critical review of world policies for mental healthcare for children and adolescents. Curr Opin Psychiatry. 2007;20:349–52.
12. Belfer, M. L., & Saxena, S. (2006). WHO Child Atlas Project. The Lancet, 367, 551–552.
13. Weiss B, Dang HM, Trung LT, Sang DL, Ngo VK, Pollack A, Tran CV, Tran NT, Do KN. A model for sustainable development of child mental health infrastructure in the LMIC world: Vietnam as a case example. Int Perspect Psychol Res Pract Consult. 2012;1(1):63–77.
14. Kleintjes S, Lund C, Flisher AJ, MHaPP Research Programme Consortium. A situational analysis of child and adolescent mental health services in Ghana, Uganda, South Africa and Zambia. Afr J Pyschiatry. 2010;13:132–9
15. Omigbodun, O. (2008) Developing child mental health services in resource-poor countries. International Review of Psychiatry, 20, 225–235.
16. WHO. (2003) Caring for Children and Adolescents with Mental Disorders: Setting WHO Directions. World Health Organization, Geneva, Switzerland
17. Burns, B.J. and R.M. Friedman, Examining the research base for child mental health services and policy. Journal Of Mental Health Administration, 1990. 17(1): p. 87-98.
18. Callaghan, J.E., L.C. Fellin, and F. Warner-Gale, A critical analysis of Child and Adolescent Mental Health Services policy in England. Clinical Child Psychology And Psychiatry, 2017. 22(1): p. 109-127.
19. Heflinger, C.A. and P.R. Dokecki, The Use of Mental Health Standards in Child and Adolescent Programs: What Factors Influence Policy Development and Implementation?, 1985.
20. Kapócs, G. and P. Balázs, The health policy in child and adolescent psychiatry in Hungary - A review of recent developments. New Medicine, 2017. 21(1): p. 14-20.
21. Kišūnaite, A. and D. Pūras, Towards a holistic approach to children's rights in Lithuanian mental health policy: A case study. Archives of Psychiatry and Psychotherapy, 2016. 18(2): p. 40-47.
22. Kutcher, S., M.J. Hampton, and J. Wilson, Child and Adolescent Mental Health Policy and Plans in Canada: An Analytical Review. Politiques et plans de sant&#x00E9 mentale pour les enfants et les adolescents au Canada : une revue analytique., 2010. 55(2): p. 100-107.
23. Lourie, I.S. and M. Hernandez, A Historical Perspective on National Child Mental Health Policy. Journal of Emotional & Behavioral Disorders, 2003. 11(1): p. 5.
24. Waddell, C., et al., Research use in children's mental health policy in Canada: Maintaining vigilance amid ambiguity. Social Science & Medicine, 2005. 61(8): p. 1649-1657.
25. Knitzer, J., Mental health services to children and adolescents: A national view of public policies. American Psychologist, 1984. 39(8): p. 905-911.
26. Braddick, F., Carral, V., Jenkins, R., & Jane-Llopis, E. (2009). Child and Adolescent Mental Health in Europe: Infrastructures, Policy and Programmes. Luxembourg: European Communities.
27. Vanesa Carral Bielsa , Fleur Braddick , Eva Jané-Llopis , Rachel Jenkins & Dainius Puras (2010) Child and Adolescent Mental Health Policies, Programmes and Infrastructures across Europe, International Journal of Mental Health Promotion, 12:4, 10-26
28. Jess P. Shatkin, Neaka Balloge, Myron L. Belfer. Child and adolescent mental health policy worldwide: an update. International Psychiatry. 2008; 5(4): 81-84
29. Waddell C, Offord DR, Shepherd CA, et al. Child psychiatric epidemiology and Canadian public policy-making: the state of the science and the art of the possible. Can J Psychiatry. 2002;47(9):825– 832
30. Jenkins R, MelzerBrian H, Jacobs B, Mcdaid D. Child and adolescent mental health: infrastructure, policies and practices in England: the CAMHEE project. *Journal of public mental health* (2010) 9(1):26-39
31. Maccoby, E.E., A.J. Kahn, and B.A. Everett, The role of psychological research in the formation of policies affecting children. American Psychologist, 1983. 38(1): p. 80-84.

**Appendix 2 Challenges for MLICs’ policy development and implementation: codes and themes**

**Theme One: Public awareness and political willingness**

**Codes:**

1. Invisible magnitude of CAMH problem
2. Distraction from political, economic and other health challenges
3. Weak and fragmented advocacy

**Theme Two: Stigma against mental illnesses**

**Codes:**

1. Stigma among patients and their families
2. Stigma among health professionals
3. Stigma among policy makers

**Theme Three: Culture values**

**Codes:**

1. Children as chattel
2. Developmental nihilism
3. Schools suffice for child development
4. CAMH as a luxury
5. Medicalization
6. Children as an economic investment
7. Respect for children and adolescents’ rights

**Theme Four: CAMH research**

**Codes:**

1. Lack of epidemiological data
2. Difficulty of obtaining data of CAMH services
3. Lack of evidence for effective treatment and intervention
4. Lack of policy and program evaluation

**Theme Five: CAMH resources**

**Codes:**

1. Lack of human resources
2. Inadequate capacity and training of human resources
3. Lack of infrastructure
4. Lack of financial resources

**Theme Six: Support from international organizations and non-governmental organizations**

**Codes:**

1. Reduction of local governments’ responsibility of CAMH
2. Fragmentation of CAMH planning
3. Unsustainability of CAMH program and funding

**Theme Seven: Others**

**Codes:**

1. Lack of policy feasibility
2. Lack of policy sustainability

**Appendix 3 Global experience and lessons for CAMH policy: codes and themes**

**Theme One: Redefining the perception of CAMH**

**Codes:**

1. Parity between mental and physical illnesses
2. Universalizing mental health for everyone
3. Socioeconomic term
4. Positive mental health

**Theme Two: Encouraging a stand-alone CAMH policy and budget**

**Codes:**

1. Government commitment to CAMH
2. Avoiding mixed budget
3. Overall framework for inter-sectorial collaboration

**Theme Three: Involving stakeholders**

**Codes:**

1. Full involvement of stakeholders
2. Negotiation and consensus
3. Involving children and adolescents as agents of action

**Theme Four: Reinforcing the role of Research and researchers in policy process**

**Codes:**

1. High-quality data and evidence of different types
2. Local experience and expertise
3. Policy advocacy
4. User-friendly communication of research
5. Partnership between researchers and policymakers
6. Relationship between CAMH with other key issues

**Theme Five: Innovating resource usage**

**Codes:**

1. Optimal use of available resources
2. Capacity building for all relevant service providers and service users
3. Cooperation with all relevant sectors and systems

**Theme Six: Maximizing positive influence of IOs and NGOs**

**Codes:**

1. Country obligations to international treaty
2. Coordination between NGOs themselves with local governments
3. Consideration of sustainability
