# Supplementary material for: Child and Adolescent Mental Health Policy in Low- and Middle-Income Countries: Challenges and Lessons for Policy Development and Implementation
Source: Front Psychiatry. 2020 Mar 18;11:150. doi: 10.3389/fpsyt.2020.00150 (PMC7094177; doi:10.3389/fpsyt.2020.00150)
Supplement: Supplementary file 2 [file Data_Sheet_2.docx]

**Supplementary material:**

**The exemplifying multi-country survey to cross-check systematic review**

**Survey design:**

We designed an exemplifying multi-country survey to cross-check findings of systematic review. The survey, based on a convenient sampling, was conducted between February and March 2019 among students from LMICs who were enrolled in the Ph.D. course of Social Epidemiology in Xiangya School of Public Health, Central South University. A questionnaire (Appendix I), consisting of three open-ended questions, was disseminated via email to the students, asking challenges or barriers for developing and implementing CAMH policy in the respondent’s country. Respondents were requested to collect information for the survey questions through all potential sources, including their government websites and documents, literature, key informants in their countries, and their own experience and judgment. Respondents were also required to provide general information of themselves, information sources for their answers, a full list of existing CAMH policies and statistics of CAMH services in their countries. Answers to the open-ended survey questions were analyzed with thematic synthesis.

**Survey results:**

In the survey, questionnaires were sent to 7 students from 6 countries. We received 5 valid questionnaires, on CAMH policy in China, Ghana, Mongolia and Sierra Leone. The two other students from Malawi and South Sudan claimed to have difficulty in providing data.

All of the respondents were previously or currently employed by government or hospitals or non-government organizations (NGOs) or public health related industry in their own countries (Appendix II). Their answers to survey questions were based on government website/document review (5 of 5), literature review (4 of 5), their own knowledge (3 of 5) and key informants (1of 5).

All respondents reported that no specific (stand-alone) CAMH policy existed in their countries. As demonstrated in Table 1, inadequacy CAMH resources, including human resources and service facilities, were found in the four LMICs. In addition, the reporting of China and Mongolia also demonstrated the unavailability of CAMH services data. For challenges or barriers of CAMH policy development and implementation, the respondents listed six (Table 2), which were consistent with the results of systematic review.

**Table 1. CAMH services in four LMICs**

|  | **China** | **Ghana** | **Mongolia** | **Sierra Leone** |
| --- | --- | --- | --- | --- |
| Child psychiatrists / psychologists | <500 by 2017 | 0 | Unknown | 0 |
| CAMH outpatient facilities | Yes* | 0 | Unknown | 0 |
| CAMH inpatient facilities | Yes* | 0 | Unknown | 0 |
| CAMH beds | Yes* | 15 by 2012 | Unknown | 0 |
| In-country child and adolescent psychiatry training programme | Yes | No | No | Yes |
| School psychological counselors | Yes* | No | Unknown | No |
| Mental health training for school teachers | Yes | No | Yes | No |

* without national statistics

**Table 2．Challenges/barriers for CAMH policy development and implementation listed by respondents from four LMICs**

| **Challenges/barriers** | **Countries** | **Quotes** |
| --- | --- | --- |
| 1. Public awareness and political willingness | China, Ghana, Mongolia, Sierra Leone, | "Delays in the approval of the Legislative Instrument (referring to Mental Health Act 2012 in Ghana) by the Attorney”  “there is poor mental health literacy among the public (in China)” |
| 1. Stigma against mental disorders | China, Sierra Leone | "a teacher told us how she was sometimes discriminated for working at a “Special Needs School (in Sierra Leone)"  “The society (in China) holds discrimination and prejudice against patients with mental disorders, which include fear, embarrassment, anger, rejection or avoidance of patients with mental disorders…” |
| 1. Biased culture value | Sierra Leone | “the cause of the (mental) sickness is not physical but spiritual/supernatural (in Sierra Leone)” |
| 1. The lack of CAMH data and research | China, Sierra Leone | “the data of CAMH problems is old and not comprehensive (in China)” |
| 1. The lack of CAMH resources | China, Ghana, Mongolia, Sierra Leone | “the lack of knowledge and skills on mental health of the primary health care providers…Psychosocial health care is poor (in Mongolia)”  “There is a lack of CAMH professionals…inadequate number and unsatisfactory capacity of full-time psychological counselors in schools (in China) ”  “Inadequate financial resources for implementation (Mental Health Act 2012 in Ghana); inadequate human resources; inadequate infrastructure for mental healthcare provision (in Ghana)” |
| 1. Sustainability of CAMH program | Sierra Leone | “the sustainability and coverage of mental health care services (i.e. mental health program based on King's Sierra Leone Partnership), including services for children, remain a challenge (in Sierra Leone)” |

**Appendix I Questionnaire of multi-country survey**

**Challenges of child and adolescent mental health policy (CAMHP) in middle and low income countries**

**Questionnaire**

**Section A. General Information of Respondent**

Name of country:

Name of the respondent:

Title/Position:

Organization/institute:

**Section B. Information sources**

Your answers to the following questions are based on which information sources: (multiple choices)

1. Government website/document browse (2) Literature review (3) Your own knowledge

(4) Key informants, please list their names and positions:

(5) Other information sources, please list

**Section C. Current status of CAMHP**

1. In your country, is there CAMHP, of any type or at any level of government? (1) Yes (2)No

**If yes to Question 1, please move to Question 2.**

**If no to Question 1, please move to Question 5.**

1. Please make the full list of your country’s CAMHPs and provide relevant information in the following table.

| **Policy document** | **Policy level*** | **Policy focus** | **Category**** |
| --- | --- | --- | --- |
|  |  |  |  |
|  |  |  |  |
|  |  |  |  |
|  |  |  |  |

* At what level has the policy been officially approved and adopted? (e.g. Cabinet, Ministry of Health, the department of mental health, Minister of Health).

**The following categorization is set according to the criteria proposed in Shatkin J, Belfer M. The global abscence of child and adolescent mental health policy. *J Child Adolesc Ment Health* 2004; 9: 104–08.

**A**- policies and/or plans that recognize the unique mental health and developmental problems of children and adolescents and clearly enumerate a unifying plan of action.

**B**- policies and/or plans that recognize the unique mental health and developmental problems of children and adolescents but do not enumerate a unifying plan of action.

**C**- policies and/or plans that recognize the mental health problems of adults and are likely to have some direct or indirect beneficial impact upon the mental health of children and adolescents.

**D**- No clearly identifiable policies or plans for mental health.

**Section D. Challenges and Barriers for CAMHP development and implementation**

1. **If yes to Question 1,** when developing the aforementioned CAMHPs, what obstacles had your country or government ever encountered?

(Based on existing literature, challenges and barriers may include but not limited to poor awareness, low political willingness, stigma, the lack of CAMH data, the shortage of CAMH resources, biased culture values, fragmented planning. Please illustrate according to your country’s situation.)

1. **If yes to Question 1,** when implementing the aforementioned CAMHPs, what challenges has your country or government ever encountered?

(Based on existing literature, challenges and barriers may include but not limited to poor awareness, low political willingness, stigma, the lack of CAMH data, the shortage of CAMH resources, biased culture values, fragmented planning. Please illustrate according to your country’s situation.)

1. **If “no” to Question 1,** has your government ever considered or will your government consider developing a CAMHP? (1) Yes (2)No

**If yes to Question 5**, please describe the situation, including motivation for policy development, policy category and focus, and potential obstacles.

**If no to Question 5**, please describe the reasons, especially the obstacles.

**Section E. Current status of child and adolescent mental health services**

1. Please provide relevant information of child and adolescent mental health resources in the following table.

| **Child and adolescent mental health resources** | **Total** | **Per 100'000 population** |
| --- | --- | --- |
| Child psychiatrists | (by year: ) | (by year: ) |
| Child psychologists | (by year: ) | (by year: ) |
| Outpatient facility specifically for children and adolescents (including services for developmental disorders) | (by year: ) | (by year: ) |
| Other outpatient services for children and adolescents (e.g. day care) | (by year: ) | (by year: ) |
| Inpatient facility specifically for children and adolescents | (by year: ) | (by year: ) |
| Child and adolescent specific inpatient beds | (by year: ) | (by year: ) |
| Schools set child psychologists (with/without certification) | (by year: ) | (by year: ) |

1. Does your country have an in-country child and adolescent psychiatry training programme? (1) Yes (2)No

**If yes to Question 7**, does the programme lead to a certificate of specialization? (1) Yes (2)No

1. Do school teachers receive any training in mental health? (1) Yes (2)No

**Appendix II Previous or current employment information of survey respondents**

1. Isaac Yaw Massey, from Ghana, was previously employed as a supervisor in Anglogold Ashanti Malaria Control program/Global Fund. Currently, he is a Ph.D. student of Department of Occupational and Environmental Health, Xiangya School of Public Health, Central South University, China.

Email: [mriymassey@ymail.com](mailto:mriymassey@ymail.com)

1. Kwabena Acheampong, from Ghana, is employed as Head of Public Health & Rehabilitation Center, Valley View University Hospital. He is a Ph.D. student of Department of Epidemiology & Health Statistics, Xiangya School of Public Health, Central South University, China.

Email: [acheampongk@aua.ac.ke](mailto:acheampongk@aua.ac.ke)

1. Oyun-Erdene Nergui, from Mongolia, was previously employed as Public Health Advisor in“New Road” Child and Family Development Center (NGO). Currently, she is a Ph.D. student of Department of Social Medicine and Health Management, Xiangya School of Public Health, Central South University, China.

Email: [oceni_17@yahoo.com](mailto:oceni_17@yahoo.com)

1. Joseph Benjamin Bangura, from Sierra Leone, is employed as Public Health Superintendent, Directorate of Primary Health Care, Ministry of Health and Sanitation. He is a Ph.D. student of Department of Epidemiology & Health Statistics, Xiangya School of Public Health, Central South University, China.

Email: [jbangura64@yahoo.com](mailto:jbangura64@yahoo.com)

1. Ouyang Feiyun, from China, was previously employed as an officer for clinical training and ethical issues in Hunan Provincial Maternal and Child Health Hospital. Currently, she is a Ph.D. student of Department of Epidemiology & Health Statistics, Xiangya School of Public Health, Central South University, China.

Email: [feiyun0716@foxmail.com](mailto:feiyun0716@foxmail.com)
